# Exploring the transcriptional and post-transcriptional regulatory networks of methionine sulfoxide reductase B1 in breast cancer

**DOI:** 10.1016/j.gendis.2026.102279

**Published:** 2026-06-06

**Authors:** Jingsong Cheng, Diyuan Zhang, Xue Chen, Xinyi Zhu, Siqi Luo, Nanting Chen, Ziheng Zheng, Qingyao Yin, Liming Yi, Na Sun

**Affiliations:** a Department of Medical Oncology, Sun Yat-sen University Cancer Center, Guangzhou, Guangdong 510275, China; b State Key Laboratory of Oncology in South China, Collaborative Innovation Center for Cancer Medicine, Sun Yat-sen University Cancer Center, Guangzhou, Guangdong 510275, China; c Department of Breast and Thyroid Surgery, Southwest Hospital, Army Medical University, Chongqing 400038, China; d The Second Clinical College, Chongqing Medical University, Chongqing 400016, China; e Department of Hematology, The Second Affiliated Hospital of Chongqing Medical University, Chongqing 400016, China; f Department of Human Anatomy, Hunan University of Medicine, Hunan 418000, China; g Key Laboratory of Chongqing Health Commission for Minimally Invasive and Precise Diagnosis and Treatment of Breast Cancer, Chongqing 400016, China

Methionine sulfoxide reductase B1 (MSRB1) is the only selenocysteine-containing enzyme in the methionine sulfoxide reductase family and repairs oxidized methionine residues.^1^^,^^2^ Our recent study identified MSRB1 as the most prominent selenoprotein in breast cancer (BRCA): it shows frequent copy-number gain, is overexpressed in tumors, and promotes proliferation, migration/invasion, and resistance to oxidative stress *in vitro*.^3^ At the upstream level, CpG methylation sites at the MSRB1 promoter have been discovered in BRCA cells,^4^ whereas in colorectal cancer, Krüppel-like factor 5 (KLF5) has been identified as a transcriptional regulator of MSRB1 recently.^5^ However, the integrated transcriptional and post-transcriptional mechanisms driving MSRB1 in BRCA remain undefined. To address this gap, we systematically charted upstream determinants of MSRB1 dysregulation in BRCA by integrating bioinformatics resources, and now present a comprehensive multi-omics map of these regulatory layers, focusing on promoter methylation (Fig. 1B), transcriptional factors (TFAP2C and TCF7L2 with EP300/KMT2C; Fig. 1C), m6A features centered on YTH N6-methyladenosine RNA binding protein F1 (YTHDF1) and A-to-I RNA editing mediated by adenosine deaminase 1 (ADAR1) (Fig. 1D), and a miR-101-3p-dependent ceRNA axis (Fig. 1E). The upstream exploration might provide direction for more effective MSRB1-based treatment strategy in BRCA.

**Figure 1:**
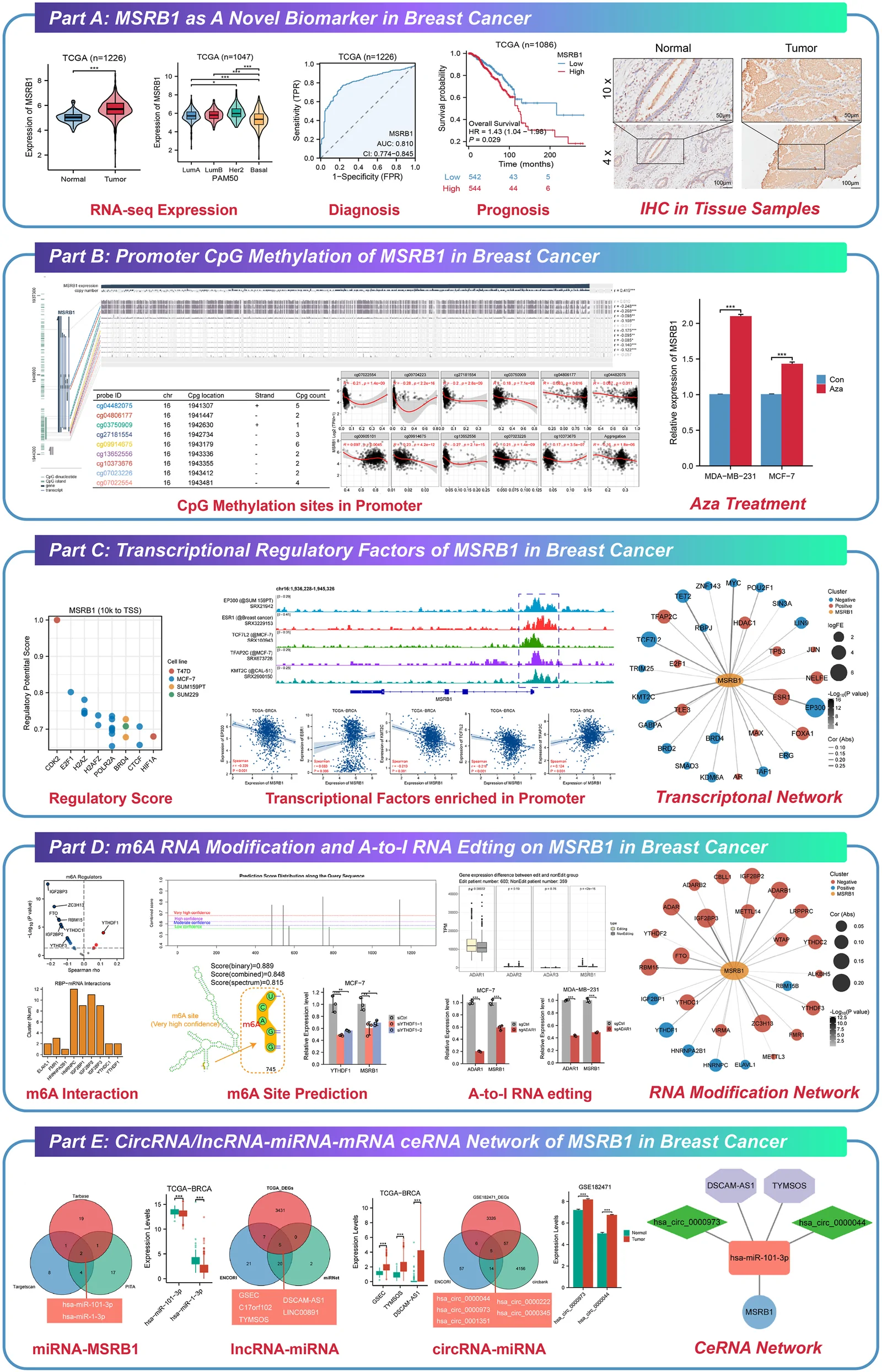
Transcriptional and post-transcriptional regulation of MSRB1 in breast cancer. **(A)** The clinical and prognostic significance of MSRB1 in breast cancer, highlighting its overexpression in tumors and its association with poorer patient outcomes. RNA-sequencing expression of MSRB1 in tumor versus adjacent normal (left); expression across PAM50 subtypes; ROC curve for tumor–normal discrimination with AUC and 95% CI; Kaplan–Meier overall survival comparing high versus low MSRB1; representative immunohistochemistry fields of tumor and normal breast tissue. **(B)** Promoter CpG methylation of MSRB1 in breast cancer, specifically focusing on the inverse relationship between promoter CpG methylation and gene expression, validated by pharmacological demethylation. Locus-level CpG atlas around the promoter (left); probe-level correlation of CpG β-values with MSRB1 mRNA expression (middle); MSRB1 mRNA after 5-azacytidine treatment in MCF-7 and MDA-MB-231 (right). **(C)** The key transcriptional regulatory factors at the MSRB1 promoter in breast cancer, identifying crucial transcription factors (TFAP2C, TCF7L2) and co-activators (EP300, KMT2C) that drive its expression. CistromeDB regulatory-potential scores (±10 kb around TSS) prioritizing transcription factors in breast contexts (left); promoter-focused ChIP-Atlas tracks showing occupancy by TFAP2C, TCF7L2, EP300, and KMT2C together with TCGA expression scatterplots for selected transcription factors (middle); network schematic summarizing promoter co-occupancy and co-activator coupling (right). **(D)** The post-transcriptional control of MSRB1, featuring m6A modification involving YTHDF1 and A-to-I RNA editing mediated by ADAR1. Correlation and RBP-mRNA interaction between MSRB1 and m6A regulators (left); predicted m6A motifs on MSRB1 RNA secondary structure. MSRB1 mRNA upon YTHDF1 knockdown (middle-left); stratification of MSRB1 expression, ADARs expression, and survival by A-to-I RNA editing status on MSRB1 mRNA. MSRB1 mRNA upon YTHDF1 or ADAR1 depletion (middle-right); RNA-modification/editing network summarizing components (right). **(E)** The upstream regulatory components of the miR-101-3p-dependent ceRNA network, including lncRNAs and circRNAs that act as sponges, collectively regulate MSRB1 in breast cancer. Intersection plots nominating miR-101-3p as the miRNA meeting tumor–normal trend, anti-correlation with MSRB1, and prognosis criteria (left); expression and correlation summaries for lncRNAs DSCAM-AS1 and TYMSOS predicted to bind to miR-101-3p (middle); expression and correlation summaries for circRNAs hsa_circ_0000973 and hsa_circ_0000044 with a schematic ceRNA model (right).

We first validated MSRB1 as a robust clinical biomarker in BRCA. Its mRNA level is significantly higher in tumors than in adjacent normal breast tissue, providing strong diagnostic discriminatory power with high ROC performance across various clinical subsets (Fig. 1A, expression/diagnosis; Fig. S1) and showing consistent up-regulation in pan-cancer and cell-line analyses (Fig. S7A and S7B). Elevated MSRB1 expression also significantly correlates with poorer overall survival (Fig. 1A, prognosis; Fig. S2), a finding robustly validated across multiple independent GEO cohorts (Fig. S7C and S7D). Immunohistochemistry further confirmed increased MSRB1 protein in BRCA tissue (Fig. 1A, immunohistochemistry and Table S1). These clinical observations provided the foundation for further mechanistic investigation.

At the DNA level, our analysis of promoter methylation, utilizing SMART, MEXPRESS, and SurvivalMeth, revealed hypermethylation around the MSRB1 promoter in tumors compared with normal breast tissue (Fig. S3A). Promoter-proximal CpG probes showed an inverse correlation between methylation and MSRB1 expression (Fig. 1B, middle; Fig. S3B and S3C). Pharmacological demethylation with 5-azacytidine increased MSRB1 expression in MCF-7 and MDA-MB-231 cells, confirming the epigenetic contribution to its intratumoral regulation (Fig. 1B, right and Table S2). However, the tumor-over-normal elevation of MSRB1 suggests that additional regulatory layers are at play (Fig. S3D).

We next identified key transcriptional regulators by integrating BRCA ChIP-seq data from CistromeDB with ChIP-Atlas. Regulatory-potential metrics prioritized transcription factor AP-2 gamma (TFAP2C) and transcription factor 7-like 2 (TCF7L2) in breast contexts (Fig. 1C, left; Fig. S4A), and MSRB1 was consistently down-regulated following TFAP2C knockdown in external datasets (Fig. S7E). mRNA correlations in TCGA further supported a role for E2F transcription factor 1 (E2F1, positive) and hypoxia inducible factor 1 subunit alpha (HIF1A, negative) in modulating MSRB1 levels (Fig. S4B and S4E). Promoter-enriched ChIP-seq resources indicate occupancy by EP300 and KMT2C alongside these transcription factors (Fig. 1C, middle; Fig. S4D). TCGA expression association trend in the same direction (Fig. S4E), suggesting a computationally inferred shared regulatory complex that may couple enhancer activity to the MSRB1 promoter through EP300/KMT2C co-activator machinery. These predicted transcriptional regulators warrant future direct experimental validation. This integrated promoter network provides a comprehensive view of these interactions (Fig. 1C, right; Fig. S4C).

Beyond transcription, the RNA itself carries marks that influence its fate. Two key mechanisms were investigated at the post-transcriptional level. For m6A modification, MSRB1 showed high-confidence m6A sites and strong association with the YTHDF1 reader, supported by CLIP-seq occupancy (Fig. 1D, left; Fig. S5A–S5D). For A-to-I RNA editing, MSRB1 expression and patient outcome varied by editing status mediated by ADAR1 (Fig. 1D, right; Fig. S5H and S5I). Crucially, experimental silencing of YTHDF1 or ADAR1 significantly attenuated MSRB1 expression in BRCA cells, confirming their positive regulatory roles (Fig. 1D, middle/right; Fig. S5F, S5G, S5J, S5K and Table S4), consistent with analysis of external perturbation datasets (Fig. S7F and S7G). However, the precise downstream molecular consequences, such as alterations in mRNA stability or translation efficiency, remain undefined and require further functional dissection. A post-transcriptional network summarizes these findings (Fig. S5H).

We finally explored ceRNA-mediated regulation of MSRB1 mRNA. A stringent analysis intersecting TargetScan, TarBase, and PITA identified miR-101-3p as a candidate meeting key criteria: inverse tumor-normal expression, negative correlation with MSRB1, and a prognostic association (Fig. 1E, left; Fig. S6A–S6D). We further identified potential upstream non-coding RNAs, including the lncRNAs DSCAM-AS1 and TYMSOS and the circRNAs hsa_circ_0000973 and hsa_circ_0000044, all of which were up-regulated in tumors and predicted to act as sponges for miR-101-3p (Fig. 1E, middle/right; Fig. S6E–S6K). This integrated miR-101-3p-dependent ceRNA network serves as a hypothesis-generating model for the strengthened MSRB1 expression in tumors (Fig. S6L). Importantly, as this network is entirely prediction-based, the functional robustness of these ceRNA interactions *in vivo* remains to be experimentally validated.

In conclusion, our study leverages multi-layered BRCA datasets to define a coherent regulatory architecture for MSRB1. While promoter-proximal CpG methylation sites exhibit an inverse correlation with expression, our findings suggest that the overall MSRB1 overexpression in tumors is predominantly driven by enhanced transcriptional and post-transcriptional programs. Specifically, a computationally predicted promoter complex anchored by TFAP2C/TCF7L2 and co-activators EP300/KMT2C, together with experimentally validated epitranscriptomic marks driven by YTHDF1 (m6A) and ADAR1 (A-to-I editing), collectively account for the sustained MSRB1 up-regulation and its prognostic significance. Our data also point to a role for a hypothetical miR-101-3p-dependent axis, where upstream non-coding RNAs may contribute to MSRB1 expression by weakening its repression. These distinct yet interconnected layers not only provide a mechanistic explanation for MSRB1’s expression dysregulation and prognostic significance in BRCA but also offer tangible molecular targets for subsequent functional studies and the potential development of more effective MSRB1-based therapeutic strategies.

## CRediT authorship contribution statement

**Jingsong Cheng:** Writing – original draft, Methodology, Investigation, Formal analysis, Data curation, Conceptualization. **Diyuan Zhang:** Writing – original draft, Resources, Conceptualization. **Xue Chen:** Writing – original draft, Validation, Methodology, Investigation. **Xinyi Zhu:** Validation, Investigation, Formal analysis. **Siqi Luo:** Resources, Investigation. **Nanting Chen:** Validation, Data curation. **Ziheng Zheng:** Investigation. **Qingyao Yin:** Validation. **Liming Yi:** Supervision, Funding acquisition. **Na Sun:** Writing – review & editing, Supervision, Project administration.

## Ethics declaration

The studies involving humans were approved by the Ethics Committee of the First Affiliated Hospital of the Army Medical University (Approval Number: KY2020055). Informed patient consent was obtained from all individuals from whom paraffin-embedded tissues were used in this study. All research procedures are conducted in accordance with relevant ethical standards and local laws.

## Data availability

The datasets used and/or analyzed during this study are available from the corresponding author upon reasonable request.

## Funding

This research received funding from the Scientific Research Fund of Hunan Provincial Education Department (China) (No. 23B1053). The funders were not involved in the study design, data collection and analysis, publication decision, or manuscript preparation.

## Conflict of interests

The authors declared no conflict of interests.
