# Supplementary material for: Exploring the transcriptional and post-transcriptional regulatory networks of methionine sulfoxide reductase B1 in breast cancer
Source: Genes Dis. 2026 Jun 6;14(3):102279. doi: 10.1016/j.gendis.2026.102279 (PMC13740839; doi:10.1016/j.gendis.2026.102279)
Supplement: Supplementary file 1 [file mmc1.docx]

Supplementary Material

# 1 Supplementary Methods and Materials

## 1.1 RNA-seq data acquisition and normalization

Utilizing UCSC XENA, RNA-seq and matched clinical data from BRCA, together with normal samples, were obtained from The Cancer Genome Atlas (TCGA) (https://portal.gdc.cancer.gov/). We extracted transcripts per million (TPM) format data and further transformed to log2 (TPM + 1) values. Patients with incomplete clinicopathological information were excluded to ensure the robustness of subsequent analyses. CircRNA expression profiling in BRCA and adjacent non-tumor breast tissues were obtained from GSE182471 stored in Gene Expression Omnibus (GEO). Data analysis was performed with the R package “plyr” (3.7.8), ROC analysis was performed using the “pROC” (1.18.0) and visualization was done using “ggplot2” (3.3.6).

## 1.2 Survival and clinical-pathological parameters analysis

For TCGA cohort, The Kaplan-Meier plotter was used to visualize the prognostic value of MSRB1 in CRC. Samples were divided into high- and low-MSRB1 based on the median. Survivals were compared by the Cox-PH model. The “survival” package (3.3.1) was used for proportional hazards hypothesis testing and fitted survival regression, and the results were visualized with “ggplot2” (3.3.6).

## 1.3 Immunohistochemistry (IHC)

IHC analysis was conducted to assess MSRB1 expression in breast tissue samples from both tumor and normal groups. Samples were fixed in 10% neutral-buffered formalin and embedded in paraffin. 4 µm thick sections were extracted from paraffin-embedded blocks and set on glass slides. Antigen retrieval was performed to enhance antigenicity, and blocking was done to limit nonspecific binding. Sections were incubated overnight at 4°C or for a specified duration with primary antibodies targeting MSRB1 (Proteintech#No.15333-1-AP, Wuhan, China; diluted 1:00). Sections were rinsed and treated with secondary antibodies linked to a reporter enzyme. Visualization was carried out using a chromogenic substrate, resulting in a visible precipitate at the locations of antigen-antibody interaction. Hematoxylin counterstaining was conducted to enhance tissue visibility. Stained sections were analyzed under an optical microscope to evaluate MSRB1 expression levels. The specific patient information was shown in **Supplementary Table S1.**

## 1.4 DNA Methylation and Aza-treatment experiment

The MART (Shiny Methylation Analysis Resource Tool) App, available at http://www.bioinfo-zs.com/smartapp, is a web-based tool designed for analyzing DNA methylation patterns in human cancers. In this study, MSRB1 methylation levels were analyzed using DNA methylation data via the SMART (Shiny Methylation Analysis Resource Tool) App ([http://www.bioinfo-zs](http://www.bioinfo-zs/)). The prognostic significance of MSRB1 DNA methylation in CRC patients was explored using SurvivalMeth (http://bio-bigdata.hrbmu.edu.cn/survivalmeth/). MDA-MB-231 and MCF-7 cells were treated with 10 μM Aza (Sigma-Aldrich, St. Louis, MO, USA) for 72 hours, after which total RNA was extracted using Trizol (Invitrogen) for qRT-PCR analysis.

## 1.5 Cell culture and siRNA transfection

MDA-MB-231 and MCF-7 human BRCA cells were obtained from the American Type Culture Collection (ATCC, Manassas, VA, USA) and maintained in DMEM (HyClone, Logan, UT, USA) supplemented with 10 % fetal bovine serum, 100 U/mL penicillin, and 100 μg/mL streptomycin under 5 % CO2 at 37 °C. YTHDF1-targeting small interfering RNAs (siRNAs) were obtained from Gene Pharma, Inc., in Shanghai, China. The sequences are listed in **Supplementary Table S2**. For all cell culture experiments, fetal bovine serum (FBS) was obtained from PAN-Biotech (P30–3302, Aidenbach, Germany). Based on the manufacturer’s specifications, selenium was not detectable in this FBS.

## 1.6 Transfected stable cell lines

To generate ADAR1 loss cells, breast cancer cell lines (MCF-7 and MDA-MB-231) were stably transfected with ADAR1 CRISPR/Cas9 plasmid expressing single-guide ADAR1 and Cas-9 (sgADAR1) or control CRISPR/Cas9 plasmid expressing single-guide control RNA and Cas-9 (sgCtrl). Then, cells were selected with 1 μg/mL puromycin and expanded. The above plasmids were kindly provided by Prof. Zeng Tu at Chongqing Medical University.

## 1.7 Real-time fluorescence quantitative PCR (RT-qPCR)

The primers of GADPH and MSRB1 were designed by the primer 5.0. Sequences for primers for RT-qPCR were listed in **Supplementary Table S2.** Total RNAs were extracted and reversely transcribed into cDNA by HiScript Q RT SuperMix (Vazyme, Nanjing, China) for RT-qPCR. GADPH and MSRB1 expression levels were conducted using 2-ΔΔCT.

## 1.8 Chromatin immunoprecipitation sequencing (ChIP-seq) analysis

ChIP-seq datasets of BRCA cell lines and tissue samples were obtained from CistromeDB (http://cistrome.org/db/#/) and ChIP-Atlas (http://chip-atlas.org) databases. Regulatory potential scores of the transcriptional factors in BRCA cells were quantified by weighting the contribution of gene surrounding peaks enriched in MSRB1 promoter. Integrative Genomics Viewer (IGV) was used to visualize the ChIP-seq tracks focused in MSRB1 promoter region, which were stored in SRX21942 (EP300@SUM 159PT), SRX3229153 (ESR1@BRCA tissue), SRX100943 (TCF7L2@MCF-7), SRX673728 (TFAP2C@MCF-7) and SRX2900150 (KMT2C@CAL-51).

## 1.9 Analysis of m6A RNA modification and A-to-I RNA editing

Canonical m6A regulators (writers/erasers/readers) were curated from standard references, and regulator–MSRB1 associations were assessed by Spearman correlation. High-confidence m6A methylation sites of MSRB1 were predicted with SRAMP (http://www.cuilab.cn/sramp

), which also provided local secondary-structure visualization around predicted sites; site coordinates and scores are listed in **Supplementary Table S3**. RBP interactions with MSRB1mRNA were retrieved from ENCORI; A-to-I RNA editing data for MSRB1 in BRCA were obtained from REIA (http://bioinfo-sysu.com/reia/) database, editing sites on MSRB1 mRNA are summarized in **Supplementary Table S4**, and per-sample editing levels provided by REIA were used for groupwise comparisons of MSRB1 and ADARs family expression (Wilcoxon rank-sum), and for survival analyses (Kaplan–Meier and Cox-PH).

## 1.10 Identification of circRNA/lncRNA-miRNA-MSRB1 network

miRNA–MSRB1 candidates were defined as the intersection of TarBase, TargetScan, and PITA. miRNA–lncRNA pairs were collected from ENCORI and miRNet and intersected with TCGA-BRCA lncRNAs differentially expressed between tumor and normal (|log2FC|>1, p.adj<0.05). miRNA–circRNA pairs were collected from ENCORI and circBank and intersected with differentially expressed circRNAs in GSE182471 (|log2FC|>1, p.adj<0.05). Edges were retained if the miRNA negatively correlated with MSRB1 and showed an opposite survival trend to MSRB1, whereas lncRNA/circRNA positively correlated with MSRB1 and shared both the tumor–normal direction and the survival trend with MSRB1; survival associations were evaluated by Cox-PH and Kaplan–Meier analyses. Interactions passing all filters were assembled into the MSRB1-centered ceRNA network.

## 1.11 Statistical analysis

Associations between continuous variables were assessed by Pearson or Spearman correlation, depending on distribution and linearity. Group differences were tested using two-tailed Student’s or Welch’s t-test for normally distributed two-group data, and Wilcoxon rank-sum test when non-normal; for ≥3 groups, one-way ANOVA with Tukey’s post hoc or Kruskal–Wallis test with Dunn’s post hoc were applied. Normality and variance homogeneity were examined by Shapiro–Wilk and Levene/Brown–Forsythe tests. All P values were two-sided, with significance set at 0.05; multiple comparisons were adjusted by Benjamini–Hochberg false discovery rate (FDR). Analyses were conducted using R 4.1.2 and GraphPad Prism 9.

# 2 Supplementary Figures

**Figure. S1. ROC performance of MSRB1 across clinical and pathological subgroups.**

(A–E) ROC curves showing the diagnostic performance of MSRB1 in breast cancer patients stratified by T stage (A), N stage (B), M stage (C), Age (D) and clinical stage (E), comparing tumor vs. normal tissue.

**Figure. S2. Prognostic significance of MSRB1 in breast cancer within different subsets.**

(A–L) Kaplan-Meier survival curves illustrating the prognostic value of MSRB1 expression in the TCGA-BRCA cohort, stratified by various clinical and pathological characteristics including T stage (A, B), N stage (C, D), M stage (E), pathological stage (F, G), ER status (H, I), HER2 status (J), and age (K, L).

**Figure S3. MSRB1 expression dysregulation: exploration of DNA methylation.**

(A) A pan-cancer box plot showing MSRB1 promoter methylation levels in normal and tumor tissues, analyzed using the SMART. (B) Spearman correlation between MSRB1 expression and specific probe of CpG methylation sites. (C) A heatmap showing MSRB1 methylation levels across different samples(D) A comparison of methylation probe levels between tumor and normal, *p < 0.05, **p < 0.01, ***p < 0.001.

**Figure S4. MSRB1 expression dysregulation: Exploration of transcription factors (TFs).**

(A) Regulatory potential scores of main TFs for MSRB1 in BRCA cells, analyzed from the Cistrome DB. (B) The correlation between key TFs, E2F1 and HIF1A, and the expression of MSRB1 in the TCGA-BRCA cohort. (C) A network diagram illustrating the correlations between MSRB1 and its main transcription regulators. (D) ChIP-Atlas tracks displaying a transcriptional complex, including TFAP2C, TCF7L2, EP300, and KMT2C, binding to the MSRB1 locus. (E) The correlation between selected transcription factors (EP300, ESR1, KMT2C, TCF7L2, TFAP2C) and MSRB1 expression.

**Figure S5. MSRB1 expression dysregulation: Exploration of mRNA modification.**

(A) Volcano plot showing the Spearman correlation between MSRB1 expression and m6A regulators in BRCA; significant regulators are labeled. (B) Prediction score distribution of putative m6A sites along the MSRB1 mRNA sequence. (C) Secondary structure visualization of the high-confidence m6A site (Position 745) predicted by SRAMP. (D) Bar chart showing the number of RBP-mRNA interaction clusters for MSRB1 retrieved from ENCORI. (E) An integrated correlation network linking MSRB1 with its potential post-transcriptional regulators, including m6A writers/readers/erasers and RNA editing factors. (F–G) Experimental validation of YTHDF1-mediated regulation in vitro. Relative mRNA expression levels of YTHDF1 and MSRB1 were measured by qRT-PCR in MCF-7 (F) and MDA-MB-231 (G) cells following transfection with YTHDF1-targeting siRNAs (siYTHDF1-1, siYTHDF1-2) compared to negative control (siCtrl). (H) Kaplan-Meier survival analysis comparing Overall Survival (OS) between BRCA patients with (group=edit) and without (group=nonEdit) MSRB1 A-to-I RNA editing events (P = 0.026). (I) Box plots comparing the expression levels of ADAR1 (ADAR), ADAR2 (ADARB1), ADAR3 (ADARB2), and MSRB1 between the edited (yellow) and non-edited (blue) groups in the TCGA-BRCA cohort. (J–K) Experimental validation of ADAR1-mediated regulation in vitro. Relative mRNA expression levels of ADAR1 and MSRB1 were measured by qRT-PCR in MCF-7 (J) and MDA-MB-231 (K) cells following CRISPR/Cas9-mediated depletion of ADAR1 (sgADAR1) compared to control (sgCtrl). Data in (F, G, J, K) are presented as mean ± SD. **P < 0.001, *P < 0.01 (Student’s t-test).

**Figure S6. MSRB1 expression dysregulation: Exploration of ceRNA regulation.**

(A–D) Plots showing the prediction and validation of upstream miRNAs of MSRB1, including their expression, correlation with MSRB1, and prognostic significance. (E–H) Plots showing the prediction and validation of lncRNAs (DSCAM-AS1, TYMSOS) that form a ceRNA with MSRB1. (I–K) Plots showing the prediction and validation of circRNAs (hsa_circ_0000973, hsa_circ_0000044) that form a ceRNA with MSRB1. (L) A schematic of the integrated ceRNA network linking lncRNAs/circRNAs, miRNA, and MSRB1.

**Figure S7. Additional multi-cohort validation of MSRB1 dysregulation, prognostic value, and upstream regulation.**

(A) Pan-cancer expression profile of MSRB1 in the TCGA cohort. Box plots compare MSRB1 mRNA levels between tumor and adjacent normal tissues across various cancer types. The Breast Invasive Carcinoma (BRCA) cohort is highlighted (red dashed box), showing significant upregulation in tumors compared to normal tissues (P < 0.001). (B) MSRB1 expression ranking across human cancer cell lines. Data derived from the Human Protein Atlas (HPA) indicates high relative abundance of MSRB1 in breast cancer cell lines (highlighted), supporting its functional relevance in this lineage. (C–D) Validation of prognostic significance in independent cohorts. (C) Forest plot summarizing univariate Cox proportional hazards regression analysis of MSRB1 in multiple independent GEO breast cancer datasets (GSE12093, GSE20685, GSE45255, GSE48390, GSE9893). Outcomes include Disease-Free Survival (DFS), Overall Survival (OS), and Relapse-Free Survival (RFS). (D) Corresponding Kaplan-Meier survival curves for the datasets listed in (C), confirming that high MSRB1 expression is consistently associated with poor prognosis. (E–G) Validation of upstream regulatory mechanisms using external perturbation datasets. Bar charts show the impact of knocking down predicted regulators on MSRB1 mRNA expression. (E) Decreased MSRB1 expression following TFAP2C knockdown in breast cancer cells (GSE126894). (F) Decreased MSRB1 expression following ADAR (ADAR1) knockdown (GSE147487). (G) Decreased MSRB1 expression following YTHDF1 knockdown (GSE148542). Data are presented as mean ± SD. Statistical significance was determined using Student’s t-test.

# 3 Supplementary Tables

## Table S1 Details for samples of immunohistochemical staining

| **Patients** | **Number** | **Group** | **Age** | **Molecular Subtype** | **Histological Type** | **Ki67** |
| --- | --- | --- | --- | --- | --- | --- |
| 1 | 8003772208 | Tumor | 40 | LuminalA | Invasive ductal carcinoma | 40% |
| 2 | 8003629657 | Tumor | 36 | Her2+ | Invasive ductal carcinoma | 60% |
| 3 | 8003920236 | Tumor | 67 | LuminalB | Non special type invasive cancer | 60% |
| 4 | 858135127 | Normal | 31 | - | Cyclomastopathy | - |
| 5 | 8003608902 | Normal | 27 | - | FB | - |
| 6 | 8001681890 | Normal | 48 | - | FB | - |

## Table S2. Sequences for siRNAs and primers for RT-qPCR

| **Oligo name** | **Oligo sequence (5’-3’)** |
| --- | --- |
| **siRNA target oligo** |  |
| siYTHDF1-1 | GATACAGTTCATGACAATGA |
| siYTHDF1-2 | GAAACGTCCAGCCTAATTCT |
| **PCR primers** |  |
| MSRB1-F | GACGTTACACCCTCACCTT |
| MSRB1-R | AGCTACTTCCGCACAGATT |
| YTHDF1-F | ATGTCGGCCACCAGCGTGGACA |
| YTHDF1-R | TCATTGTTTGTTTCGACTCTGC |
| ADAR1-F | CATCAGCGGGCTGTTAGAAT |
| ADAR1-R | CTTGGCCACTTTCTTGCTTC |
| GAPDH-F | CAGTGCCAGCCTCGTCCCGTAGA |
| GAPDH-R | CTGCAAATGGCAGCCCTGGTGAC |

## Table S3. Precited m6A methylation sites of MSRB1 mRNA

| **ID** | **Position** | **Score**  **(binary)** | **Score**  **(knn)** | **Score**  **(spectrum)** | **Score**  **(combined)** | **Decision** |
| --- | --- | --- | --- | --- | --- | --- |
| 1 | 484 | 0.77 | 0.302 | 0.837 | 0.773 | m6A site  (Very high confidence) |
| 2 | 543 | 0.756 | 0.529 | 0.82 | 0.77 | m6A site  (Very high confidence) |
| 3 | 572 | 0.65 | 0.526 | 0.489 | 0.579 | m6A site  (Low confidence) |
| 4 | 747 | 0.889 | 0.668 | 0.815 | 0.848 | m6A site  (Very high confidence) |
| 5 | 779 | 0.643 | 0.458 | 0.483 | 0.57 | m6A site  (Low confidence) |
| 6 | 1141 | 0.836 | 0.627 | 0.826 | 0.821 | m6A site  (Very high confidence) |

## Table S4. A-to-I RNA editing sites of MSRB1 mRNA in TCGA-BRCA

| **id** | **chr** | **position1base** | **strand** | **ref** | **ed** | **sample** | **cytoband** | **geneRegion** | **location** | **repeat** |
| --- | --- | --- | --- | --- | --- | --- | --- | --- | --- | --- |
| 1 | chr16 | 1936149 | - | T | C | 10 | 16p13.3 | intergenic | ALU | SINE/  AluSx |
| 2 | chr16 | 1936175 | - | T | C | 18 | 16p13.3 | intergenic | ALU | SINE/  AluSx |
| 3 | chr16 | 1936237 | - | T | C | 16 | 16p13.3 | intergenic | ALU | SINE/  AluSx |
| 4 | chr16 | 1936382 | - | T | C | 223 | 16p13.3 | intergenic | REP | LTR/  LTR42 |
| 5 | chr16 | 1936439 | - | T | C | 294 | 16p13.3 | intergenic | REP | SINE/  FLAM_C |
| 6 | chr16 | 1936454 | - | T | C | 208 | 16p13.3 | intergenic | REP | SINE/  FLAM_C |
| 7 | chr16 | 1936453 | - | T | C | 331 | 16p13.3 | intergenic | REP | SINE/  FLAM_C |
| 8 | chr16 | 1936526 | - | T | C | 120 | 16p13.3 | intergenic | REP | SINE/  FLAM_C |
| 9 | chr16 | 1936701 | - | T | C | 333 | 16p13.3 | intergenic | ALU | SINE/  AluSz6 |
| 10 | chr16 | 1936700 | - | T | C | 321 | 16p13.3 | intergenic | ALU | SINE/  AluSz6 |
